# Evaluation of an ultrasound program in nationwide Continuing Professional Development (CPD) in Korean public health and medical institutions

**DOI:** 10.1186/s12909-022-03271-4

**Published:** 2022-04-10

**Authors:** Claire Junga Kim, Hyojung Mo, Ji Young Lee

**Affiliations:** 1 Department of Medical Humanities, Dong-A University College of Medicine, Busan, Korea; 2 Education & Training Centre for Public Healthcare, National Medical Centre, Seoul, Korea; 3 Clinical Research Coordinating Center, Seoul St. Mary’s Hospital, The Catholic University of Korea, Seoul, Korea

**Keywords:** Program evaluation, CIPP (context, Input, Process, Product) evaluation, Continuing professional development (CPD), Nationwide educational program, Public hospital

## Abstract

**Background:**

The Education and Training Centre for Public Healthcare of the National Medical Centre plays a key role in providing continuing professional development (CPD) to 221 public health and medical institutions in South Korea. To assess the realization of the Centre’s core value and the intended changes, program evaluations are required. The context, input, process, and product (CIPP) model is particularly suitable for evaluating CPD in the public sector, as it allows for recognizing the dynamic nature of the program environment.

**Methods:**

This research applied the CIPP model to the evaluation of CPD programs, particularly abdominal and thoracic ultrasound programs implemented in 2017 and 2018. Data were collected from 2017 to 2019. The program and its feedback were reviewed in the context evaluation. Based on this, a subsequent program strategy was established for the input evaluation. Observing the program in real time and recording its progress was followed in process evaluation. Finally, the outcomes and impacts of the program were reviewed and compared with baseline data in the product evaluation.

**Results:**

In context evaluation, the educational needs of the Centre’s CPD program recipients, impediments that inhibit participation in education, and resources that the Centre can utilize were identified through an online survey, focus group interviews and expert consultation. Through input evaluation, we identified the best alternative that satisfied all pre-selected criteria, which were responsiveness to priority system needs, potential effectiveness, fit with existing services, affordability, and administrative feasibility. Observing the program in real time and recording its progress were conducted in process evaluation, demonstrating that the augmented program went as planned, and even had to be expanded due to increased demand. The impact of the program was measured, interpreted, and assessed in the product evaluation. The review committee decided that the intended change had been occurred, thus the Centre decided to maintain the program.

**Conclusion:**

A thorough evaluation is necessary to determine the potential benefits of CPD. The CIPP methodology is valuable for executing formative and summative evaluations. The CIPP model is particularly useful for securing accountability data for large-scale nationwide educational programs supplied by public funds.

**Supplementary Information:**

The online version contains supplementary material available at 10.1186/s12909-022-03271-4.

## Background

Continuing professional development (CPD) for physicians is important to achieve safe, effective, and improved clinical care [1]. However, the potential of CPD programs is not fully realized as they tend to deliver arbitrary content rather than to meet practical educational needs, selecting teaching methods based on educator convenience such as lectures or observation. Much needed emphasis is now being placed on gap analysis [2], targeting practice changes [3–5], and adult education practices [6]. A thorough evaluation of existing programs is the first step for applying innovative CPD approaches in educational practice.

The Education and Training Centre for Public Healthcare at the National Medical Centre (NMC), hereafter referred to as the Centre, plays a key role in providing CPD to 221 public health and medical institutions (PHMIs) in South Korea. PHMIs are established and operated by the state, local governments, and public institutions [7]. The Centre is responsible for providing CPD to 57 PHMIs that fall under the supervision of the Ministry of Health and Welfare, comprising over a quarter of the total PHMIs in the country and 2121 physicians, as of December 2018, thereby making up 17.1% of the total number of physicians in Korean public healthcare hospitals. Due to its sheer reach, the Centre is one of the most influential institutions in the public healthcare sector.

The Centre’s core values are to focus on enhancing publicness in healthcare and improving the quality of medical services [8]. Enhancing publicness in healthcare entails strengthening the role of PHMIs to fulfil unmet needs in the healthcare system, addressing areas where the free market typically fails (i.e., infectious disease outbreaks, natural disasters, or emergencies).

Program evaluations should assess how well the Centre’s core values are realized and whether the intended changes have occurred. Educational programs are fundamentally focused on change; therefore, program evaluations should assess whether the desired changes occur as a result of educational initiatives [9]. The CPD setting calls for specific criteria that the program must fulfill. For example, since CPD is funded by public money, accountability is particularly important along with potential effectiveness and administrative feasibility. In addition, it is important to assess whether the needs and objectives of the program are clear, as well as whether the program is tailored to meet these needs and objectives. The Centre’s programs are required to prepare physicians for public health roles regardless of their prior residency training or specialty, to be capable of treating the majority of patient groups including chronic disease patients, and to be prepared to meet public healthcare demand in times of disaster.

Among the diverse methodologies [9–11], the context, input, process, and product (CIPP) model has the most suitable characteristics for the evaluation of CPD in the public healthcare sector, which is affected by external and internal factors, as well as factor interactions. Situations such as the outbreak of new infectious disease or natural disasters are what physicians in the public healthcare sector need to be trained for. They should also be able to respond to increased need for the treatment of chronic disease patients and issues, such as an aging population. Diverse participants and stakeholders, governmental and related ministries, that operate programs, program policies, and the calls from civil society also affect educational programs. These factors influence each other. For these reasons, public health CPD should be treated as a nonlinear, complex, and dynamic system [12, 13].

Informed by the complexity theory [14, 15], the CIPP model recognizes the importance of clinical and educational contexts and accommodates numerous uncertainties within the educational program [9]. The CIPP model allowed us to recognize “the dynamic, septic condition of the real world” ([16] p. 351), and at the same time meticulously considers the program environment and multiple inputs to the program, as is essential in the evaluation of public health CPD. Additionally, the CIPP model can provide both formative and summative evaluations [16, 17]; therefore, the model’s strength lies in monitoring implementation and measuring improvements during and after implementation, typically on a yearly basis. Moreover, as CPD programs are funded by taxpayers, they must be held accountable. CIPP is particularly useful in this regard because all data involving strategy planning, provision, and monitoring of education, as well as objective assessments, are translated into accountability data. Therefore, the CIPP model is suitable for use in the evaluation of CPD by the Centre.

## Methods

We applied the CIPP model to evaluate CPD programs implemented by the Centre in 2017 and 2018. The Centre offered 17 programs in 2017 and 10 programs in 2018. Among these programs, we evaluated abdominal and thoracic ultrasound programs because major changes were made to these programs, in terms of strategy, educational goals, content, and methodology in 2018, following a needs assessment in 2017, which showed the strongest demand for change among other programs. Educational evaluation is useful for examining the effectiveness of a program after changes are made and for determining whether to continue the changes.

To answer the evaluation questions in each phase of the CIPP model, data were collected from learners, instructors, operators, stakeholders, and external experts between May 2017 and January 2019 (Table 1). We evaluated the abdominal and thoracic ultrasound courses delivered to physicians at PHMIs working under the Ministry of Health and Welfare. This study was approved by the institutional review board of the National Medical Centre, Seoul, Korea.

**Table 1:** Questions used to evaluate CIPP components and data collection methods^*^

CIPP components	Evaluation questions	Data collection method
Context	What is necessary or useful: in other words, what are the educational needs? What are the impediments to meeting necessary or useful needs? What pertinent expertise, services, or other assets are available? What relevant opportunities (e.g., funding opportunities, administrative support) exist?	- Document review - Literature review - Demographic data analysis - Surveys - Records analysis (e.g. test results, learner performance data) - Focus groups - Advisory group
Input	What are the potential approaches to meeting the identified educational need? How feasible is each of the identified approaches, given the specific educational context of the need? How cost-effective is each identified approach, given the specific educational context of the need?	- Literature review - Expert consultants - Inviting proposals from persons interested in addressing the identified needs - Pilot trials to assess available human and material resources to evaluate the work plan and strategy for relevance, feasibility, cost, and economy
Process	How was the programme actually implemented, compared to the plan? Are/were programme activities on schedule? If not, why? Is/was the programme running on budget? If it is/was over or under the planned budget, why? Is/was the programme running efficiently? If not, why? What do/did participants and observers think about the quality of the process?	- Participant observers - Document review - Open-ended survey questions provided to the participants (learners, operators, instructor) - Periodic exchange of information with project leaders and staff to monitor and provide feedback on the process and record the actual process
Product	What positive outcomes of the programme can be identified? What negative outcomes of the programme can be identified? Were the intended outcomes of the programme realised? Were there unintended outcomes, either positive or negative? What are the short-term implications of programme outcomes? What are the longer-term implications of programme outcomes? How effective was the program? How sustainable are the intended and positive programme outcomes?	- Stakeholders’ judgments of the project or programme (Evaluation from the education and training review committee) - Comparative studies of outcomes with those of similar projects or programmes (Including expert evaluation) - Assessment of achievement of programme objectives (Usefulness at work, academic achievement, etc.) - Surveys (Level of satisfaction) - Participant reports of project effects (Self-evaluation) - Comparing outcomes to assessed needs (Comparative Studies of outcome with assess needs)

^*^Adopted from Stufflebeam’s original suggestion on data collection methods (Stufflebeam 2003) and evaluation questions to CIPP evaluation studies (Frye and Hemmer 2012, p. 296)

We used the CIPP methodology as an ongoing evaluation tool. The ultrasound program and its feedback were reviewed in 2017 through context evaluation. Based on this, the 2018 program strategy was established through input evaluation. The planning and implementation of the program were assessed through process evaluation. Finally, the outcomes and impact of the 2018 program were reviewed and compared through product evaluation with baseline data from the context evaluation. Both formative and summative evaluations were performed using the CIPP model. The formative evaluation was executed by two of the authors, JL and HM, who participated as operators in continuous monitoring. The summative evaluation was executed by all three authors, including CJK, who participated in the record analysis as an external expert after the completion of the program in both 2017 and 2018.

### Context evaluation

Educational needs, impediments, opportunities, assets, and resources, such as expertise and services, should be included in the context evaluation. To assess the educational needs of potential participants in CPD programs, we conducted an online survey targeting 1010 physicians at 39 PHMIs, among which 204 physicians replied, thereby yielding 178 valid responses. We conducted focus group interviews (FGIs) from December 5, 2017, to March 8, 2018, to achieve in-depth interpretations of the survey results. A total of 106 valid responses were received from the 111 participants.

The FGIs targeting learners followed a democratic view of educational needs assessment approaches [18]. This approach is advantageous because it involves numerous stakeholders in goal-setting, thereby allowing them to determine the relative importance of potential needs. However, this approach may have uncovered participants’ preferences, rather than their actual needs, thus we did not rely solely on learner FGIs. The same research questions were posed to an expert advisory group to ensure that the potential shortcomings associated with FGI responses can be complemented by informed analytical perspectives [18]. In addition, document review, literature review, demographic data analyses of learners, and 2017 program record analyses were included in the contextual evaluation.

### Input evaluation

In the input evaluation, the most cost-effective and feasible strategy was selected to best satisfy the needs identified through context evaluation. Our online survey responses and FGI-based needs assessment indicated that the ultrasound program was most in need of improvement. Therefore, we performed an input evaluation for the ultrasound program. A literature review, expert consultation, and stakeholder workshops were conducted to identify the strategy selection criteria [17]. Upon completion of the 2017 program, three external experts reviewed the records, assessed the outcomes and impacts, and made suggestions for improvement. Twenty-eight directors of public hospitals also participated in the stakeholder workshop and developed the strategy selection criteria for the 2018 program.

### Process evaluation

Process evaluation was conducted by providing an ongoing assessment of the plan implementation, followed by documentation of the process [17]. Acting as process evaluators, two of the authors compared the initial plan for the program and actual practice, identified the reasons for the differences, and monitored feedback for each participant. Real-time monitoring and documentation followed. Additionally, an open-ended survey was administered to learners, instructors, and operators after the completion of each program module.

### Product evaluation

The goal of product evaluation is to measure, interpret, and assess the outcomes and impacts of a program [17]. Upon the completion of each education module, participant satisfaction and reports of project effects on outcomes and impacts were surveyed. Academic achievement was assessed at the end of the module and compared with pre-test results. An online survey assessing how well the learning was applied at worksites was conducted 3–6 months after the program completion. As stakeholders, an education and training review committee (directors of PHMIs and medical education experts) evaluated the 2018 program and compared the results with the 2017 data. The CIPP methodology examines all outcomes related to education, from the intended positive impacts and short-term results to unintended impacts and long-term results [9]. Therefore, open-ended questions were included in the survey conducted after completion of the program to assess unintended outcomes.

## Results

### Context evaluation

In the context evaluation, the educational needs of the recipients of the Centre’s CPD program, impediments inhibiting participation in education, and resources that the Centre can utilize were identified. Our online survey results were analyzed for educational needs assessment (Table 2). Due to the low number of respondents, the online survey could not present a definitive conclusion regarding the current educational needs. Nevertheless, we considered this data important because those who answered are potential learners with educational aspirations. Moreover, issues with low response rates can be supplemented by other methods, such as FGI. Approximately half of the 178 respondents (49.1%) reported that they chose to participate in educational programs based on the usefulness to their work.” Therefore, developing practical CPD programs was an important task. When asked about what makes it difficult to apply learning to the work environment, 38.4% selected “difference in hospital system,” and 36.5% selected “lack of manpower and equipment.” This finding indicates that educational programs not considering learners’ working environments can become obstacles. In addition, 38.1 and 35.3% selected “no one to cover the work while absent” and “inconvenient location,” respectively, and 78.5% of the respondents reported preferring 1 day, instead of one night and 2 days, as an appropriate program period.

**Table 2:** 2017 Educational needs assessment: Online survey conducted after the completion of the 2017 programme (Top five, listed in order)^*^

Questions/ rank	1	2	3	4	5
Q. What factors do you consider when choosing a course?	Usefulness at work (*n =* 135, 49.09%)	Location (*n =* 61, 22.18%)	Instructors (*n =* 36, 13.9%)	Duration (*n =* 30, 10.91%)	Cost (*n* = 12, 4.36%)
Q. What are the barriers to your participation of the course?	No one to cover work (*n* = 98, 38.13%)	Inconvenient location (*n* = 36, 35.29%)	Lack of information (*n* = 48, 18.68%)	Low budget for education assistance (*n* = 16, 6.23%)	Indifference of management (*n* = 11, 4.28%)
Q. What are the challenges when applying course materials in real work environment?	Difference in hospital systems (*n* = 98, 38.43%)	Lack of manpower and adequate equipment (*n* = 93, 36.47%)	Heavy workload (*n* = 43, 16.86%)	Uncooperative colleagues (*n* = 12, 4.71%)	Indifference of management (*n* = 4, 1.57%)
Q. What do you think the most optimal duration of the training programme is?	1 day (*n* = 62, 39.24%)	2 days 1 night (*n* = 62, 39.24%)	3 days 2 nights (*n* = 24, 15.19%)	more than 5 days (*n* = 4, 2.53%)	4 days 3 nights (*n* = 2, 1.27%)

^*^The total number of respondents was 204, but 178 responses were analysed excluding missing data. Online surveys with structured questionnaires were conducted and self-administered by respondents

Similar results were derived using FGIs, which were conducted to achieve an in-depth interpretation of the online survey results (Appendix). Many respondents pointed out barriers to participating in the educational program, such as the absence of staff who can cover for them at work and geographical and temporal accessibility. Poor advertising of educational programs and a workplace atmosphere that discouraged participation in education were also noted among the responses. These responses clearly indicate many barriers for participation in educational programs. Instead, increasing the temporal and geographical accessibility of programs and making education mandatory have been suggested as factors that could lead to higher participation.

The ultrasound educational program was the course that respondents most wished to take because it was the most applicable in primary medical care. However, the ultrasound program was cancelled several times in 2017 because of a lack of enrolment. This indicates that the content and delivery of education can prevent learners from enrolling in a program, despite high awareness of its utility, which is problematic.

Whether it is applicable in the practical field is also a key factor prominent in FGIs. The groups, who responded to the overall curriculum in 2017 were good but not well-applied, pointed out that they could not use their learning in the practical field due to differences between the environment of a hospital in Seoul and a remote local hospital. The environment, human resources, facilities, and even patient groups are vastly different from that of a university hospital in Seoul and a local hospital in remote areas. In addition, interest and practical applications are prominent factors in promoting learners’ participation. Therefore, to recruit physicians as potential learners and draw good responses, educational content that can be applied in the environment where they work must be developed.

In consultation with experts, expanding accessibility and providing educational content tailored to the roles played by public health institutions in the community were highlighted. In particular, significant gaps were observed in the actual clinical environment and patient groups between the learners and university hospital where the educational program was outsourced.

In the literature review, two goals for PHMIs were revealed as immediate challenges: ameliorating the financial independence of hospitals by securing sufficient profits and pursuing public interest in improving the health of the local community [19–21]. The fact that PHMIs must achieve these goals simultaneously was highlighted in 2013 through the closure of a century-old PHMI, Jinju Medical Centre, which caused significant social repercussions [22].

In addition, labor productivity was found to greatly influence public hospital profits. Among them, the labor productivity of physicians accounted for a significant part of PHMI’s labor productivity [21]. Due to the lack of physicians, only one doctor was placed in-charge for each department in most PHMIs. The directors of PHMIs may not be favorable toward physicians attending educational programs due to the financial losses caused by the lack of physicians. Considering these findings together, it was concluded that physicians cannot attend long-term educational programs due to the current situation in hospitals. Based on the online survey results, FGIs, and expert consultations, the ability to quickly identify emergency patients or treat elderly patients with chronic conditions was especially important in learners’ clinical environments. The location of the program and program length were also critical.

The abdominal and thoracic ultrasonography program was determined to require the most changes in needs assessment due to the largest gap between willingness to attend the program and actual participation rate. Moreover, the Centre can potentially be held accountable for cancelled programs if poorly planned during inspections by the government administration. Therefore, we reviewed records from the 2017 educational program. Initially, the Centre planned to deliver three ultrasonography courses for a total of 10 classes. However, only six classes were completed, which leads to a potential accountability issue. Financial support from the Ministry of Health and Education and administrative support from the Centre were secured for the program.

### Input evaluation

Through input evaluation, we identified the best alternative that satisfied all preselected criteria. After consultation with external experts and workshops with stakeholders, responsiveness to priority system needs, potential effectiveness, fit with existing services, affordability, and administrative feasibility were selected as criteria. The abdominal and thoracic ultrasound program was maintained in 2018 because it fulfilled the priority system needs, thereby enabling the quick screening of emergency patients. However, it was clear that the program required improvement. We selected educational materials and providers through open competitive bidding by using a targeted outsourcing strategy for the abdominal and thoracic ultrasound course only. This strategy was determined to be superior to the outsourcing strategy used in 2017 when a total of 17 CPD programs were outsourced *en bloc* to a single contractor. While the Centre provided administrative support, it selected one academic society in a related field that best fulfilled the criteria.

One academic society fulfilled the criteria of responsiveness to priority system needs and fit with existing services by setting the educational goal as differentiation of emergency diseases and achievement of skills required for quick referral in primary care. It also fulfills the criteria for potential effectiveness by establishing a practice-centric educational method. By shortening the educational period from four nights and 5 days in 2017 to one night and 2 days, we satisfied the criteria of potential effectiveness and fit with existing services. The educational program described in the initial proposal was affordable and administratively feasible. For these reasons, the academic society was selected as the provider of educational content. In addition, considering the demand for improved geographical accessibility, we decided to provide education not only in Seoul, but also in other areas.

### Process evaluation

After adopting these changes, the revised abdominal and thoracic ultrasound program was introduced in 2018. This program proceeded as planned and even had to be expanded due to increased demand. The initial goal was to educate 24 clinicians in two sessions with 12 students each. However, due to the increased accessibility of the program, compared with the 2017 program in terms of time and location, more students signed up than expected. More sessions were scheduled, thus bringing the program operation rate to 150% and the education recipient rate to 187%, with both being higher than those in 2017 (Table 3).

**Table 3:** Programme plan and outcomes for 2018 programm

	Plan	Outcome	Achievement Rate
Number of Courses Provided (module)	2	3	150%
Number of Participants (n)	24	45	187.5%
Duration of Course in Days (D)	2 Days	2 Days	–
Duration of Course in Hours (H)	16	15.8	97.8%

Two of the authors observed the sessions in real-time and recorded progress. The learners, instructors, and operators were requested to complete the free-answer questionnaires. The data obtained were then analyzed during a meeting at the Centre, where concerns were raised that the necessary capabilities may not have been sufficiently trained due to the shortened education period. However, the responses from learners during the sessions indicated that they were not only satisfied but also quite confident about their learning. The instructors believed that the learners had demonstrated the required level of competence. Freely-answered questionnaire responses were collected from all instructors, learners, and operators. The instructors said that the program was highly effective, citing intense concentration and enthusiasm among the learners. The learners were satisfied with the learning content that could be applied in daily clinical practice and the diverse case studies they were provided. Many learners left comments thanking the instructors and operators. The operators described the vibrant interactions between the instructors and learners.

### Product evaluation

**Fig. 1:**
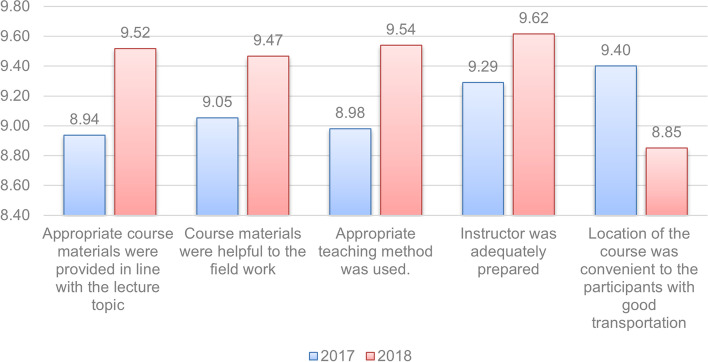
Program satisfaction levels for 2017 and 2018

Goal attainment was assessed by examining post-education improvements in academic achievement. When comparing pre- and post-education academic achievements, the scores for abdominal ultrasound course and thoracic ultrasound course increased from 3.04 to 7.75 and from 3.07 to 8.06, respectively. Academic achievement, measured based on self-efficacy, increased from an average of 3.06 out of 10 to an average of 7.9 post-education. The educational satisfaction levels for the 2017 and 2018 programs (Fig. 1) showed that the 2018 program scored higher than the 2017 program, in terms of topic satisfaction, teaching method, applicability, and instructor preparedness. In terms of geographic accessibility, the 2018 program scored lower than the 2017 program. However, respondents who considered the four nights and 5 days program held in Seoul in 2017 to be too long and distant did not participate in the first place, and only those for whom it was easy to attend participated, the 2017 participants may have been quite satisfied with the geographic accessibility of the program location. Based on an analysis of per capita education costs, the 2018 program accounted for only 84.1% of the total investment in the 2017 program.

Three to 6 months after the completion of the program, the recipients were asked if they had experienced progress in their performance by applying what they learned in their work. On a scale of 1 to 10, the average score was 7.19. An obstetrician-gynecologist shared a story in a newsletter about how he successfully improved the diagnosis and survival rates of emergency patients after receiving ultrasound education. All short- and long-term effects were examined and reflected in the decision-making process. An education and training review committee consisting of officials from the Ministry of Health and Welfare, public medical institution directors, medical education experts, and physicians conducted the evaluations and made decisions as stakeholders. The review committee pointed out that only a small number of students could receive ultrasound education because of the high cost of the program. Still, the committee agreed that the content of the program corresponded with the goals of public medical institutions and the Centre’s core values, and thus evaluated the modifications carried out in 2018 to be effective for fulfilling educational goals. Accordingly, they decided to maintain the program, which was provided in 2019.

## Discussion

In this study, the CIPP model was applied to evaluate CPD programs targeting physicians employed by public health institutions in Korea. CPD provided to physicians working in the public healthcare sector has significant potential to directly improve the quality of community healthcare. To realize the potential of CPD to the fullest, program evaluations must be conducted thoroughly, and improvements to educational programs must be based on such evaluations. The CIPP method makes it possible to highlight the context in which CPD is provided, which in our case was public healthcare and physicians in the public healthcare sector. In context evaluation, we emphasized the following factors: education is funded by taxpayer money; education should be geographically and temporally accessible to doctors working in diverse environments and facilities; education should rely on administrative support from the Centre. Therefore, during the input evaluation, we adopted an education strategy that fulfilled the criteria of being responsive to priority system needs, potential effectiveness, fit with existing service, affordability, and administrative feasibility. Improvements were made accordingly, and enhanced abdominal and thoracic ultrasound courses were provided in 2018. Through process and product evaluation, we demonstrated that the 2018 program had a higher number of participants, compared to that of the previous year (2017), as well as higher levels of participant satisfaction and cost-effectiveness.

This study has limitations because it analyzed only one program provided by the Centre (which provided a total of 17 courses in 2017 and 10 courses in 2018) over a short span of time. Educational programs are constantly affected by factors within and outside the program. Therefore, it is clear that numerous programs provided by the Centre must have mutually affected each other. Needs assessment was carried out through surveys and FGIs, with the subject being the entire educational program provided by the Centre.

The scope of this study was limited to an ultrasound educational program that was identified as a high priority through needs assessment. Selecting one course for the program cannot expand its positive effects to the entire program (10 courses in 2018). In addition, this narrow scope may have made it impossible to analyze the complexity and interrelations between programs. Moreover, the FGIs conducted in 2017 were based on voluntary participation, thus only 10% learners participated in the FGIs. Therefore, we cannot dismiss the possibility of bias toward the inclusion of enthusiastic learners. Long-term outcomes, such as the impact on patient treatment, were not evaluated. The study was based on data collected May 2017 to January 2019, when the 2017 program was initiated to when the applications of practical skills were surveyed for the 2018 program. Therefore, the long-term outcomes or impacts of the program have not been fully examined. Nonetheless, one obstetrician-gynecologist reported improvements in the diagnosis and survival rates of emergency patients after receiving ultrasound education.

Despite the limitations mentioned above, the study has strengths in being comprehensive, having conducted evaluations at all phases beginning with the previous educational program, improvement and implementation of the revised program, and evaluation of the revised program after implementation. In addition, ongoing program evaluation is carried out based on a shared understanding, thus determining whether year-long education is being provided as planned can be reviewed later. We assessed not only whether the intended outcomes were achieved, but also how and why they were achieved, thus the process can be reproduced in the future, and accountability data can be secured in the future as well.

## Conclusions

The CIPP approach is suitable for the evaluation and improvement of CPD in public health. It enables the analysis of interactions between internal and external factors affecting educational programs, as well as the context in which the program is provided. Therefore, the CIPP approach helps to produce desired changes in educational programs and evaluate and improve programs funded by taxpayer money because accountability data are generated while simultaneously executing formative and summative evaluations. CPD programs administered by the Centre have the potential to improve quality of care provided by physicians in Korean PHMIs, and eventually to enhance public health. The educational program we examined exhibited notable improvement after the application of the CIPP approach, thus we were successful in realizing our goals.

## Supplementary Information

**Additional file 1.**

## Data Availability

The datasets generated and analyzed during the current study are not publicly available due to ethical requirements at the National Medical Centre. However, the educational programs and annual evaluation reports are available from the corresponding author.

## Abbreviations

CIPP: Context, Input, Process, and Product.
CPD: Continuing professional development
NMC: National Medical Centre
PHMIs: Public health and medical institutions
