# Supplementary material for: Evaluation of an ultrasound program in nationwide Continuing Professional Development (CPD) in Korean public health and medical institutions
Source: BMC Med Educ. 2022 Apr 10;22:261. doi: 10.1186/s12909-022-03271-4 (PMC8996612; doi:10.1186/s12909-022-03271-4)
Supplement: Supplementary file 1 — Additional file 1. [file 12909_2022_3271_MOESM1_ESM.docx]

Appendix. Interviews with physicians to develop the training and education program

| Theme | Sub-theme | n | Quotation 1 | Quotation 2 |
| --- | --- | --- | --- | --- |
| 1. Experiences in previous NMC educational programmes | (1) Good and well applied | 25 | The education I received helped me in my clinical practice because the well-organised programme sufficiently satisfied my educational needs. In fact, the application of the educational material to practice was plausible in the field. | Experiences in the educational programme were pleasant and valuable as the course offered plenty of time to practice with a small number of people. |
|  | (2) Good but not well applied | 25 | Theory and practice were two different things. The educational programme might have been beneficial to some degree, but there were considerable differences between hospitals in Seoul and rural areas. The latest medical trend and knowledge learned from the course were unable to apply to this local hospital as it can be in Seoul. | Applying what I learned during the programme in practice was difficult as a result of substantial limitations in the facilities and environment of the local hospital. |
|  | (3) Not good and not well applied | 5 | The educational programme did not offer what local hospitals need and instead offered only what the educational institutions could provide. | One of the prime concerns was how the educational programme could be incorporated into the local medical centre, and as expected, a gap between theory and practice was uncovered. |
|  | (4) Did not know / did not attend | 45 | This is the first time I have heard of this course. | I have never participated in the course as I have never received an official letter or notice of invitation. |
|  |  |  |  |  |
| 2. Barriers to participation in the education program | (1) Staff absences | 59 | The director of the hospital is reluctant to permit doctors to attend the educational programme as it leads to staff absences. | Because the hospital has only one doctor for each department, absences due to study leave without any backup are practically impossible. |
|  | (2) Geographical accessibility | 14 | Due to the long distance to be travelled, attending the educational programme in Seoul is unrealistic, especially for doctors from remote areas. | Since geographical accessibility is one of the most critical priorities for doctors from rural areas, operating educational programmes by region is crucial, so education centres should be established in locations students can easily access. |
|  | (3) Temporal accessibility | 21 | Because other conferences are also held during the weekends, attending the course on weekends is rather achievable. | Most previous educational programmes were on weekdays, so adjusting the schedule was very complicated. Attending a four-night and five-day course is impractical and unreasonable. |
|  | (4) Lack of publicity | 26 | The educational programme at the National Medical Centre (NMC) is outstanding. However, strengthening public relations is required to promote the program. | I had no idea that the programme provides the Continuing Medical Education (CME) credits accredited by Korean Medical Association (KMA).  Moreover, education expenses and room and board are covered. Looking at it now, the educational content and support are excellent. |
|  | (5) Ignorance/non-acceptance by leaders | 9 | The director of the hospital does not feel the need for further educational programmes. The director’s opinion is that a specialist does not need more education in clinical practice. | The director is cynical about physicians attending educational programmes for more than two days. |
|  |  |  |  |  |
| 3. Participation promoting factor | (1) Mandatory education | 17 | In terms of profitability, the hospital’s perspective is that it is a loss for a doctor to attend educational programmes. If a penalty for low participation is enforced, hospital management typically decides to allow physicians to attend the programmes. | Enforcing a rule that requires doctors to attend essential educational programmes should be launched. NMC should ensure that further education is mandatory so the management would have no choice but to allow it. |
|  | (2) Temporal accessibility and  replacement workforce availability | 70 | I normally attend general department conferences for one-day training sessions. The main reason is that adjusting the schedule during the week is too complicated. | The Ministry of Health and Welfare should secure a pool of medical doctors and provide workforce support when local hospital doctors must receive training. |
|  | (3) Institutional cooperation, CME credit, hospital evaluation, and incentives | 15 | Since the NMC is involved in dictating medical centres’ budgets, providing incentives or budget support for each medical centre depending on the training participation rate would be a good strategy. | Supporting education expenses and accommodation required by trainees is an efficient way to increase participation rates. |
|  | (4) Interest, practical application | 69 | The Ministry of Health and Welfare and the NMC should work together to see the big picture. The analysis of data by region, patient, and doctor must be conducted before analysing what kind of education is needed. | The educational programme should provide information at the general hospital level rather than in too much detail or on unusual cases. |
|  | (5) Geographical accessibility | 27 | Accessibility is important because many medical professionals cannot travel extensive distances. | Regional educational programmes should be launched. |
|  | (6) Online class | 11 | Developing and distributing online and mobile educational content like other educational institutions would be helpful. | Online education should address the latest medical trends and knowledge. |
| ­ | (7) Increase in publicity | 14 | I was unaware of things such as the CME credit accredited by the KMA and the support for room and board expenses. Promoting these aspects of the educational programme seems necessary. | Other medical educational programmes regularly send academic journals and e-mails every month. The CME programmes should also be promoted in this manner. |
|  |  |  |  |  |
| 4. Course you want to take | (1) Ultrasound | 19 | Since many procedures are performed using ultrasound, launching an ultrasound course would be beneficial. | Ultrasonic procedures require continuous education because they are part of primary medical care. |
|  | (2) Emergency treatment | 6 | I urgently need training in the complex skills required in the emergency room. | We are a small hospital with a shortage of staff. As a urologist, I find it challenging to prepare for emergencies, so I need further training. |
|  | (3) Geriatric medicine and chronic disease (hypertension, diabetes, etc.) | 6 | Patients in the hospital are mostly older with chronic conditions (hypertension, diabetes, chronic lung disease, etc.). Receiving general training to determine at what point and at what level to transfer patients from primary and secondary hospitals to tertiary hospitals is required. | Introducing educational programmes on geriatric medicine would be helpful because our region has a large elderly population. |
